# The Microbiota–Gut–Brain Axis: Key Mechanisms Driving Glymphopathy and Cerebral Small Vessel Disease

**DOI:** 10.3390/life15010003

**Published:** 2024-12-24

**Authors:** Che Mohd Nasril Che Mohd Nassir, Muhammad Danial Che Ramli, Mazira Mohamad Ghazali, Usman Jaffer, Hafizah Abdul Hamid, Muhammad Zulfadli Mehat, Zaw Myo Hein

**Affiliations:** 1 Department of Anatomy and Physiology, School of Basic Medical Sciences, Faculty of Medicine, Universiti Sultan Zainal Abidin, Kuala Terengganu 20400, Terengganu, Malaysia; nasrilnassir@unisza.edu.my (C.M.N.C.M.N.); mazira.mohamadghazali@gmail.com (M.M.G.); 2 Faculty of Health and Life Sciences, Management and Science University, Shah Alam 40150, Selangor, Malaysia; muhddanial_cheramli@msu.edu.my; 3 Department of Neurosciences, School of Medical Sciences, Universiti Sains Malaysia, Kubang Kerian 16150, Kelantan, Malaysia; 4 Kulliyyah of Islamic Revealed Knowledge and Human Sciences, International Islamic University Malaysia, Kuala Lumpur 50728, Malaysia; jafferu@iium.edu.my; 5 Department of Human Anatomy, Faculty of Medicine and Health Sciences, Universiti Putra Malaysia, Serdang 43400, Selangor, Malaysia; a_hafizah@upm.edu.my (H.A.H.); m_zulfadli@upm.edu.my (M.Z.M.); 6 Department of Basic Medical Sciences, College of Medicine, Ajman University, Ajman P.O. Box 346, United Arab Emirates

**Keywords:** microbiota, gut–brain axis, microparticles, cerebral small vessel disease, sleep, glymphatic system

## Abstract

The human microbiota constitute a very complex ecosystem of microorganisms inhabiting both the inside and outside of our bodies, in which health maintenance and disease modification are the main regulatory features. The recent explosion of microbiome research has begun to detail its important role in neurological health, particularly concerning cerebral small vessel disease (CSVD), a disorder associated with cognitive decline and vascular dementia. This narrative review represents state-of-the-art knowledge of the intimate, complex interplay between microbiota and brain health through the gut–brain axis (GBA) and the emerging role of glymphatic system dysfunction (glymphopathy) and circulating cell-derived microparticles (MPs) as mediators of these interactions. We discuss how microbial dysbiosis promotes neuroinflammation, vascular dysfunction, and impaired waste clearance in the brain, which are critical factors in the pathogenesis of CSVD. Further, we discuss lifestyle factors that shape the composition and functionality of the microbiota, focusing on sleep as a modifiable risk factor in neurological disorders. This narrative review presents recent microbiome research from a neuroscientific and vascular perspective to establish future therapeutic avenues in targeting the microbiota to improve brain health and reduce the burden of CSVD.

## 1. Introduction

The human microbiota represent a very diverse microbial community that inhabits most, if not all, body sites; it plays a fundamental role in maintaining health and influencing disease processes. Comprising trillions of bacteria, viruses, fungi, and archaea, it participates in basic physiological processes from metabolism and immune modulation to maintaining homeostasis [1,2]. The gut microbiota have become widely studied for elaborating such a complex interaction with the host that it will have implications for gastrointestinal health and extend into systemic processes, including the brain [3].

Accumulating evidence has illustrated the role of the gut–brain axis (GBA), a bidirectional communicatory network between the gut microbiota and brain function [3,4]. The ability of microbiota to influence neuroinflammation, cognitive function, and emotional well-being is mediated through several means, namely metabolic production, immune modulation, and alteration of the gut barrier [5,6]. Currently, studies have proven that dysbiosis, or imbalance of microbial composition, is implicated in a wide range of clinical neuropsychiatric conditions such as depression, anxiety, and even autism spectrum disorders [7,8].

With the increased interest in microbiota and their involvement in neurological health, it has become very timely to research their relevance to cerebral small vessel disease (CSVD). CSVD is a prevalent cerebrovascular disorder with changes in the small, penetrating arteries supplying blood to the brain. It is associated with several adverse outcomes, such as cognitive impairment, vascular dementia, and stroke [9]. Several recent publications have shown that chronic inflammation and endothelial dysfunction, the hallmarks of CSVD, are modulated by the gut microbiota, offering a new avenue to reconsider the pathophysiology [10,11].

Recently, another critical player that helps to clear metabolic waste from the brain through a network has come to light: the glymphatic system [12]. Impaired glymphatic function (or glymphopathy) might be linked to various neurodegenerative diseases and cognitive decline [13,14]. Of note, recent findings suggest that the microbiota may indirectly modulate this process of glymphatic activity through systemic inflammation and circadian rhythm regulation, further supporting a complex association between gut health and brain function [15,16,17].

Moreover, circulating cell-derived microparticles (MPs), or extracellular vesicles, are small membrane-bound particles released from various cell types during their activation or apoptosis [18]. More recently, they have gained significant attention due to their mediating role in intercellular communication and inflammation [18]. In the context of CSVD, MPs may thus facilitate the transfer of pro-inflammatory signals from the gut to the brain, thereby potentiating neuroinflammation and endothelial dysfunction [19,20]. Furthermore, there is evidence that gut dysbiosis may be associated with the increased release of MPs, thus directly linking gut health and cerebrovascular pathology [21,22,23].

Thus, in this narrative review, we hope to discuss the complex relationship between human microbiota and neurological health, emphasising cerebral small vessel disease and the glymphatic system. By integrating the current literature on microbial dynamics, neuroinflammation, and vascular health, we aspire to explain a potential therapeutic avenue to leverage the microbiota to enhance brain health and diminish the impact of cerebral small vessel disease.

## 2. The Human Microbiota: An Overview

Human microbiota are the collective community of microorganisms, including bacteria, archaea, viruses, fungi, and protozoa, inhabiting different body sites, such as the gut, oral cavity, skin, and other mucosa. Among these, the gut microbiota are amongst the most studied; in the gut, trillions of microorganisms bear important functions related to digestion, metabolic activity, and immune response [1,2]. Each human has a microbiotic signature shaped by unique individual genetic makeup, environmental exposures, and lifestyle behaviours [24,25].

Moreover, the composition of the microbiota varies significantly among different anatomical sites. According to research from the Human Microbiome Project (HMP) [26] and the Metagenomics of the Human Intestinal Tract (MetaHIT) consortium [27], the human gut is home to 2766 microbial species. Bacteria from the phyla Proteobacteria, Firmicutes, Actinobacteria, and Bacteroidetes account for more than 90% of the gut microbiome [27,28]. The bulk of gut bacteria are Firmicutes, which include Gram-positive *Lactobacillus* species and Gram-negative *Bacteroides* species [29]. *Fusobacteria* species and *Verrucomicrobia* species account for the remaining 10% of the gut microbiome [30]. In turn, the skin microbiota are dominated by Actinobacteria, especially *Propionibacterium* and *Corynebacterium* species [31]. This diversity and abundance are critical for their functional capacity and resilience against perturbations.

Several factors affect the composition and diversity of human microbiota: geographical location, hygiene practices, and exposure to antibiotics capable of tremendous changes in microbial populations are among the leading ones. According to Ridaura et al. [32], such factors take leading positions among others. Genetic predisposition may also be considered, whereby genetic factors can influence the establishment and maintenance of gut microbiota [33,34]. Moreover, dietary pattern is among the critical determinants of microbiotic composition. Whereby diets high in fibre, for example, allow for the proliferation of beneficial microbes that can produce short-chain fatty acids (SCFAs), contributing to colonic health and regulation of the immune system [35]. Finally, lifestyle factors such as physical activity and stress modulate microbiota to promote health or disease [36].

Furthermore, the human microbiota not only allow for homeostasis and regulate the host’s immune system, but they also help digest complex carbohydrates and synthesize essential nutrients such as vitamins B and K [37]. Besides, the microbiota are crucial for the normal development and functioning of the immune system. They help the immune system to differentiate between dangerous and benign pathogens, avoiding inappropriate inflammatory responses [2,11,21]. However, dysbiosis, characterised by loss of microbial diversity and imbalance of microbial populations, has been linked to various diseases, including autoimmune disorders, allergies, and metabolic syndrome [5,38].

Hence, it is worth noting that the microbiota communicate with host cells through various signalling pathways and modulate inflammation in systemic and immune responses. Metabolite production, which encompasses SCFAs, can do more than just give energy sources to colonocytes, as they exert anti-inflammatory effects that are believed to contribute toward maintaining gut and systemic health [38]. In brief, the human microbiota represent an elaborated and dynamic ecosystem that is core in preserving health, regulating the immune system, and preventing disease. The factors that shape the composition and function of microbiota are of exceptional importance in developing strategies that will promote health and mitigate disease. These complex interactions point to the necessity of studying the interrelations of the microbiota with neurological health first and foremost through the GBA.

## 3. Microbiota and the Brain: The Gut–Brain Axis (GBA)

The GBA is a term for the bidirectional communication network between gut microbiota and the central nervous system (CNS). According to Cryan et al. [3], this bidirectional interaction is of utmost importance in maintaining homeostasis and modulates other physiological processes, including emotional regulation, cognition, and immune response. The GBA includes neural pathways, endocrine, and immune systems—the gut microbiota have a significant influence far beyond the borders of the gastrointestinal tract.

Multiple mechanisms help with communication within the GBA. These are mainly through microbial metabolites, especially SCFAs derived from gut bacteria fermentation of dietary fibres. The most common forms of SCFAs, such as acetate, propionate, and butyrate, have been demonstrated to cross the blood–brain barrier (BBB) and affect brain function, influencing neuroinflammation, neurotransmission, and neurogenesis [39]. Moreover, some gut bacteria can modulate the synthesis and functioning of neurotransmitters such as gamma-aminobutyric acid (GABA) and serotonin, which directly influence mood and behaviour [40,41].

On the other hand, the immune system is the other crucial player in the GBA. The gut microbiota play a vital role in developing and regulating the immune system, influencing systemic inflammation and the integrity of the BBB [5,6,39]. It has been demonstrated that dysbiosis increases intestinal permeability, leading to systemic inflammation, which may contribute to neuroinflammatory disorders such as depression and anxiety [4,7,41]. For example, some intestinal bacteria can induce the production of cytokines, interleukin-6 (IL-6) and tumour necrosis factor-alpha (TNF-α), which may easily cross the BBB and negatively affect neuroinflammation processes [42].

**Figure 1:**
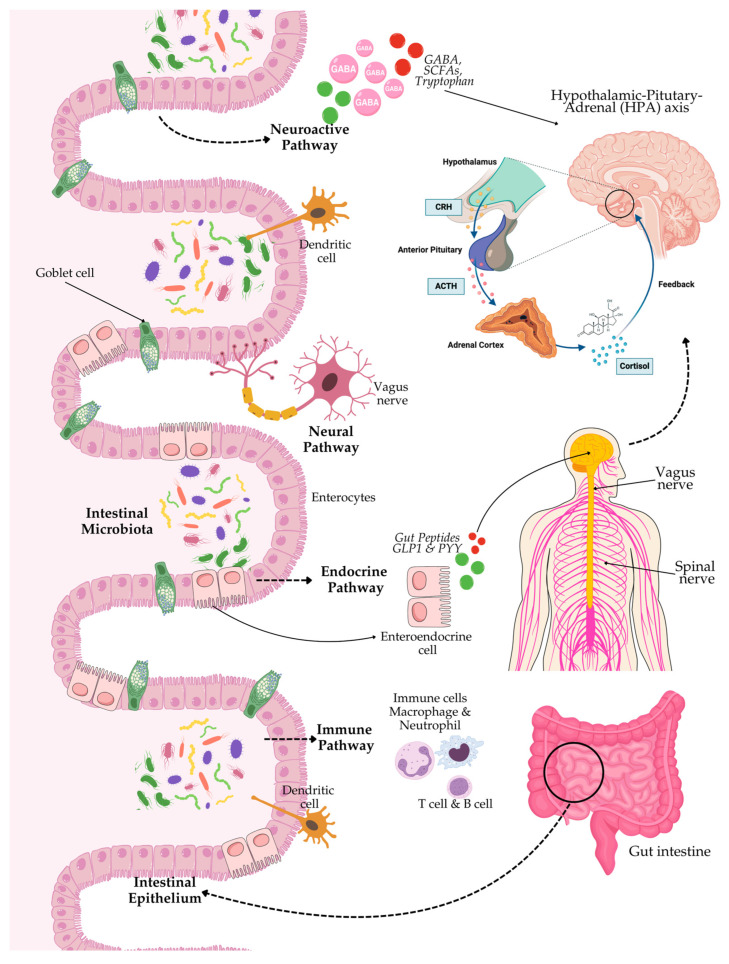
Schematic diagram of gut–brain–microbiota axis. The brain and gut microbes communicate via many pathways. The central nervous system (CNS) interacts with gut microbes through various direct and indirect gut–brain axis (GBA) pathways. They include the immune pathway (including cytokines), short-chain fatty acids (SCFAs), and microbial metabolites; the neuroactive pathway (including neurotransmitters and neuroactive metabolites); the neural pathway (including the enteric nervous system, vagus nerve, and spinal nerves); and the endocrine pathway, i.e., the hypothalamic–pituitary–adrenal (HPA) axis. The HPA axis response involves hypothalamic neurons that release hormones such as corticotropin receptor hormone (CRH) into the portal circulation of the brain, resulting in the release of adrenocorticotropic hormone (ACTH), which initiates cortisol production and release. Cortisol regulates the neuroimmune signalling reactions.

The neural pathway is another critical GBA communication factor involving the enteric nervous system (ENS), vagus nerve, and spinal nerves. The ENS has even been known as the “second brain”, with thousands of neurons coating the gut wall and capable of regulating gastrointestinal activities independently [43,44]. Besides, the vagus nerve is one of the primary conduits between the gut and the brain. The vagus nerve mediates signals from the gastrointestinal tract to the brain, consequently playing a role in the regulation of autonomic responses and the response to stress in the organism [45,46]. The endocrine pathway also plays an essential role in the GBA, mainly through the hypothalamic–pituitary–adrenal (HPA) axis (see Figure 1). The HPA axis is excited by stress, whereby hypothalamus neurons release corticotropin-releasing hormone (CRH) into the portal circulation of the brain. This release starts a chain reaction with the synthesis of adrenocorticotropic hormone (ACTH) from the pituitary and subsequent cortisol production and release from the adrenals [47] (see Figure 1). Cortisol, as a major stress hormone, represents a modulatory hormone of neuroimmune signalling reactions in as much as it can modulate immune responses and neuronal activity. These very elaborately developed communication pathways make the interaction between gut microbiota and the brain quite complex, as microbial signalling can overwhelm neurobiology and behaviour.

The influence of dysbiosis is thus profound in brain health. Emergent research alludes to the fact that an imbalance of gut microbiota can exacerbate neurodegenerative diseases, affect cognitive functions, and influence mood disorders. For example, such studies have identified that individuals with depression show altered gut microbiota profiles compared to healthy controls, thus pointing to an association between microbial composition and mood regulation [48]. Besides that, animal models of neurodegenerative diseases have shown that dysbiosis might exacerbate neuroinflammation and accelerate cognitive decline, indicating the importance of microbial health in maintaining brain function [49].

Conversely, emerging evidence points out that the liver is a crucial player in the GBA, hence establishing the gut–brain–liver axis. The liver controls metabolic homeostasis, immune responses, and detoxification, modulating gut microbiota and brain functions [50,51]. Liver dysfunction, as in disturbances of bile acid metabolism or systemic inflammation, disrupts gut barrier function and leads to neuroinflammatory responses that perpetuate neurological disorders such as Alzheimer’s disease and hepatic encephalopathy [52]. This axis is of clinical relevance and therapeutic potential given its modulation of bile acid pathways, gut microbiota, and reduction of liver inflammation with regard to both physiological and pathologic processes.

Moreover, interventions aimed at restoring a microbial balance, such as applying probiotics and dietary modification, have also taken the front seat in helping improve mental health outcomes. In some instances, clinical trials have suggested that certain types of probiotics might reduce symptoms of anxiety and depression, perhaps through changes within the GBA itself [53]. The GBA is a dynamic process between the gut microbiota and the CNS, involved in almost every physiological and psychological process. Understanding how this relationship works will be important to inform new therapeutic approaches that promote brain health and mitigate the challenges posed by dysbiosis. Therefore, with the expansive effects of gut microbiota in the brain, it is important to look into their possible roles in certain neurological conditions, such as CSVD, due to dysbiosis acting through microbial interaction contributing to neuroinflammation and vascular pathology.

## 4. Cerebral Small Vessel Disease and Microbiota

Cerebral small vessel disease (CSVD) is considered the leading cause of stroke and dementia, responsible for approximately 25% of ischemic strokes and a large proportion of vascular dementia [54]. CSVD involves small arteries, arterioles, capillaries, and brain venules, causing various pathological changes, including cerebral microbleeds, white matter hyperintensities (WMHs), lacunes, and cerebral microinfarcts [55]. These structural abnormalities often lead to clinical manifestations encompassing the impairment of cognition, disturbances in gait, and mood disorders, leading to diminished quality of life in the elderly [56].

Multiple overlapping factors make the underlying pathophysiology of CSVD multifactorial, which includes chronic endothelial dysfunction, disturbed cerebral autoregulation, thrombo-inflammation, oxidative stress, and disruption of the BBB [57]. Damage to the endothelium starts a cascade of pathological events that include increased permeability of the BBB, leakage of plasma proteins, and perivascular inflammation [58]. Moreover, chronic low-grade inflammation contributes to vascular stiffness and impairment in cerebral microcirculation, leading to subsequent ischemia, white matter degeneration, and ultimately cognitive impairment [59,60]. Furthermore, oxidative stress, through the overproduction of reactive oxygen species (ROS), exacerbates endothelial injury and promotes neurodegenerative processes that further deteriorate CSVD outcomes [59,60].

### Potential Links Between Gut Microbiota and CSVD

Recent evidence points to the potential involvement of gut microbiota dysbiosis, or imbalance in microbial homeostasis, in CSVD pathogenesis. Gut dysbiosis is characterized by increased intestinal permeability, enabling microbial endotoxins, such as lipopolysaccharides (LPS), to translocate into the systemic circulation. These endotoxins induce systemic inflammation, which can cross the BBB, thereby contributing to neuroinflammation, a key factor in developing CSVD [3].

There is also gut dysbiosis-related inflammation that contributes to the development of endothelial dysfunction and impairs the integrity of the cerebrovasculature. Systemic high levels of pro-inflammatory cytokines, for instance, TNF-α and IL-6, predispose one toward vascular stiffness, defective vasodilation, and increased vascular resistance, contributing to the small vessel injury characteristic of CSVD [61]. Besides, gut-derived metabolites, such as trimethylamine N-oxide (TMAO), have been associated with increased atherosclerotic burden and vascular inflammation, thus perhaps exacerbating cerebrovascular damage in CSVD [62].

**Figure 2:**
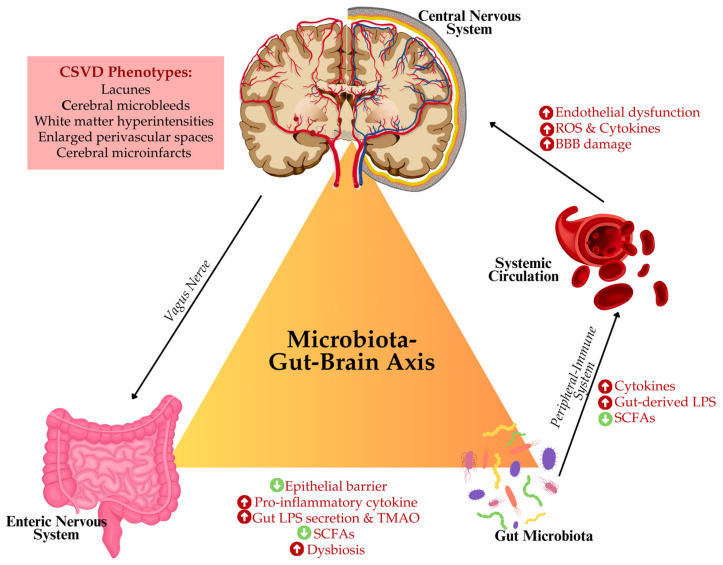
Schematic representation linking the microbiota–gut–brain axis and its potential impact on cerebral small vessel disease (CSVD). An imbalance in gut microbiota, also known as dysbiosis, contributes to decreased short-chain fatty acids (SCFAs) activity, increased secretion of lipopolysaccharides (LPS), trimethylamine-N-oxide (TMAO), and pro-inflammatory cytokines. These alterations can weaken the gut epithelial barrier and promote a systemic inflammatory response and vice versa. Dysbiosis-related molecules such as cytokines and gut-derived LPS enter the systemic circulation, activating the peripheral immune system and leading to further inflammation. These circulating factors influence the central nervous system through vagal signalling and systemic inflammation. In the brain, they contribute to disrupted microcirculation, i.e., endothelial dysfunction, increased production of reactive oxygen species (ROS), elevated cytokine levels, and blood-brain barrier (BBB) damage. This cascade of events ultimately exacerbates CSVD phenotypes by promoting neuroinflammation, vascular impairment, and potential cognitive decline.

Furthermore, gut dysbiosis also influences another significant factor in CSVD, which is oxidative stress. The imbalance in the microbiome can facilitate the excessive generation of ROS, which then result in the oxidative destruction of cerebral vasculature, impair the regulation of cerebral blood flow, and increase white matter damage [59,60]. Some gut-derived metabolites, including SCFAs, especially butyrate, exert protective functions against disturbance in endothelial function or neuroinflammation [36,39]. Figure 2 illustrates the putative concept of microbiota–GBA concerning CSVD. Thus, it is assumed that modulation of gut microbiota might be a promising therapeutic strategy for preventing or minimizing features of CSVD.

The accumulating evidence has pointed out that the gut microbiota influence not only the vasculature per se but also the brain’s waste clearance system, i.e., the glymphatic system. The glymphatic system plays an essential role in metabolic waste removal, including amyloid beta (Aβ), from the brain, processes in which disturbances have been recorded in both CSVD and neurodegenerative diseases [12]. Disruptions in the gut microbiota have been associated with impaired glymphatic function or glymphopathy, which may further promote the accumulation of neurotoxic proteins and contribute to the cognitive decline of CSVD patients [63].

Recent studies have suggested that gut microbiota-targeting interventions, including probiotics, prebiotics, and dietary approaches, may improve cerebrovascular health. Indeed, certain strains of probiotics have been shown to dampen systemic inflammation and improve endothelial function, suggesting that targeting microbiota may represent a novel therapeutic approach in CSVD treatment and prevention [53]. In summary, CSVD is a leading cause of stroke and cognitive impairment, increasingly being recognized to be influenced by gut microbiota through mechanisms that include inflammation, oxidative stress, and vascular dysfunction. Understanding the GBA and its role in cerebrovascular health opens new avenues for therapeutic interventions to modulate microbiota to prevent or mitigate the progression of CSVD.

## 5. Overview of the Glymphatic System and Its Role in Brain Waste Clearance

The glymphatic system is a specialized waste-clearing system of the brain responsible for removing metabolic byproducts, including Aβ, tau proteins, and other neurotoxic substances. It functions through perivascular channels, particularly the aquaporin-4 (AQP4) water channels polarized on the astrocytic feet, which enable the movement of cerebrospinal fluid (CSF) into the brain parenchyma for exchange with interstitial fluid and subsequent clearance of waste products [12]. This system is activated during rest, especially during slow-wave, non-rapid eye movement (non-REM) sleep, in which the brain’s interstitial space is expanded to improve the flow of CSF and the removal of wastes [64].

Alarmingly, if the integrity of the cerebrovasculature is disrupted, as it is in CSVD, this glymphatic dysfunction (or glymphopathy) may further promote the accumulation of metabolic waste products in the brain [14,65,66]. The vascular stiffness, endothelial damage, and impaired cerebral autoregulation accompanying CSVD may disorganize the clearance routes of the glymphatic system, allowing the accumulation of neurotoxic substances, such as Aβ, associated with cognitive decline and neurodegeneration [65]. Therefore, glymphopathy may thus be a contributing aspect of the disease process for both CSVD and associated neurodegenerative conditions, such as vascular dementia, Parkinsonism and Alzheimer’s disease (AD) [65,66].

### 5.1. Glymphopathy as a Catalyst for Neuroinflammation and Endothelial Damage in CSVD

Emerging evidence points to glymphopathy significantly contributing to neuropathology and endothelial damage, two significant pathologies in CSVD. Glymphopathy results in neurotoxic protein deposition within the interstitial space of the brain. For instance, Aβ has been reported to cause chronic neuroinflammation by activating microglia into the secretion of pro-inflammatory cytokines, such as IL-6 and TNF-α, which directly injure the cerebral endothelial cells. Recent studies indicate that even a 20% reduction in the rate of glymphatic clearance, i.e., ventricular CSF clearance, can induce a statistically significant temporal increase in cerebral amyloid levels and, thus, the risk of cognitive decline and neurodegeneration [67].

Moreover, the persistence of metabolic waste in glymphatic-affected regions appears to perpetuate endothelial oxidative stress, a critical factor in vascular injury. Amyloid accumulation impairs mitochondrial function in endothelial cells, leading to excessive ROS production. Increased levels of ROS promote BBB permeability, facilitating the diffusion of inflammatory cells and cytokines into brain parenchyma and further amplifying the inflammatory milieu within CSVD [59,60]. Furthermore, impaired glymphatic activity compromises interstitial fluid dynamics, influencing cerebral perfusion and promoting local hypoxia [62]. The hypoxic environment stimulates the release of vascular endothelial growth factor, which, though having a neuroprotective effect in an acute way, becomes perniciously angiogenic after chronic exposure and destabilizes endothelial integrity. A recent study further correlates glymphopathy with the significant upregulation of VEGF expression in ischemic stroke patients, further associating impaired glymphatic function with endothelial dysfunction [68].

Finally, glymphopathy promotes a pro-inflammatory state that perpetuates vessel wall thickening, arteriolosclerosis, and vessel occlusion, the characteristic features of CSVD. Indeed, the chronic inflammatory burden from impaired glymphatic flow has been linked to an increased 15% risk for white matter hyperintensities and cognitive impairment in CSVD patients independently of comorbidities such as hypertension and diabetes [69]. In summary, glymphopathy is one of the critical drivers of neuroinflammation and endothelial damage in CSVD, presenting new therapeutic targets aimed at improving glymphatic flow and dampening inflammatory cascades. Further studies into modulators of glymphatic efficiency may lead to leaps in management that are nothing short of quantum.

### 5.2. Role of Microbiota in Regulating Glymphatic Function

Recent evidence suggests that gut microbiota might influence glymphatic function by regulating systemic inflammation and circadian rhythm [17,70]. The neuroinflammatory consequences of dysbiosis-induced inflammation, especially the release of circulating LPS and pro-inflammatory cytokines, could perturb astrocytic regulation of CSF flow and promote neuroinflammation. Both factors facilitate glymphopathy [71]. Moreover, such inflammatory cytokines weaken the integrity of the BBB and hinder the glymphatic system from efficiently clearing metabolic waste.

Besides that, gut microbiota control the host’s circadian rhythms, which directly relate to the activity of the glymphatic system. The most active state of the glymphatic system occurs during sleep; disturbance in circadian rhythm regulation or sleep–wake cycles can have adverse effects on glymphatic system activities [70]. Intestinal bacteria control the circadian rhythm by producing necessary metabolites, including SCFAs and neurotransmitters like serotonin, which modulate sleep–wake cycles and promote clearance via the glymphatic system during sleep [7,71]. Dysbiosis could thus lead to disturbed sleep and, subsequently, impaired functioning of the glymphatic system, leading to the accumulation of wastes in the brain that could contribute to the lower cognitive scores seen in CSVD [72].

### 5.3. The Connection Between Sleep Quality, Glymphatic Clearance, and Microbiota

The correlation between sleep quality, glymphatic function and gut microbiota composition is becoming increasingly convincing. Poor sleep quality, sleep fragmentation, or sleep deprivation may lead to impaired glymphatic clearance of neurotoxic substances, determining the accumulation of Aβ and tau proteins implicated in neurodegenerative diseases and cognitive impairment [73]. Sleep disturbances are associated with an increased risk of CSVD, thus underlining the importance of sleep and glymphatic function in maintaining cerebrovascular health [74,75].

In addition, the composition of gut microbiota also plays a role in sleep modulation [76]. Sleep disturbance has been related to reduced diversity and enrichment of pro-inflammatory bacterial species that may foster systemic and neuroinflammation. Besides, diverse and healthy microbiota, especially the abundance of SCFA-producing bacteria, are associated with better sleep quality and higher efficiency in glymphatic clearance [76,77]. These findings suggest that gut microbiota modulation-based intervention strategies may promote sleep quality and glymphatic function, possibly providing a potential therapeutic benefit in CSVD prevention and treatment.

In summary, CSVD, a significant cause of stroke and cognitive impairment, is increasingly recognised as being influenced by the GBA, with the gut microbiota playing a pivotal role in its pathogenesis. Inflammation, oxidative stress, and vascular dysfunction—critical drivers of CSVD—are modulated by gut microbiota dysbiosis. Furthermore, emerging evidence highlights the crucial role of the glymphatic system in brain waste clearance, which is closely linked to sleep quality and gut microbiota composition. Understanding these intricate connections opens new therapeutic avenues targeting the gut microbiota to improve glymphatic function, cerebrovascular health, and cognitive outcomes in CSVD.

## 6. The Impact of Sleep and Diet on the Microbiota

### 6.1. Sleep Disruption and Its Influence on Microbiota Diversity

Sleep is one of the crucial biological processes, and its disturbance affects several physiological systems, including gut microbiota. Disturbances in the pattern of the sleep–wake cycle, fragmented sleep, or disruption of circadian rhythm affect the diversity and composition of microbiota gravely, as discussed. More than this, even one night of sleep deprivation reportedly causes low microbial diversity, i.e., increased *Firmicutes* to *Bacteroidetes* ratio, higher abundances of the families *Coriobacteriaceae* and *Erysipelotrichaceae*, and lower abundance of *Tenericutes* [73,78]. Further, it causes enhanced replication of pathogenic bacteria with a reduced abundance of beneficial species [78]. The changes in gut microbiota might induce systemic inflammation and metabolic dysregulation-insulin resistance, a factor closely related to the pathogenesis of cerebrovascular diseases, including CSVD.

Some evidence has suggested that sleep deprivation can disturb the production of microbial metabolites, including SCFAs, essential for intestinal barrier integrity and immune response modulation [79]. SCFAs, like butyrate, decrease gut permeability, prohibiting translocation into the systemic circulation of bacterial endotoxins such as LPS. Besides, lack of sleep also affects metabolism and food intake, leading to dietary changes that influence the gut microbiome. For example, people with disrupted sleep patterns often consume more sugary or high-fat foods, promoting the growth of certain bacterial species like Firmicutes over Bacteroidetes and impacting metabolic health [80,81]. Additionally, sleep disturbances could increase cortisol levels via the activation of the HPA axis, thus further disturbing gut microbiota by facilitating the growth of bacterial strains associated with inflammation [82]. Therefore, increased circulating LPS, diet-related sleep disruption and altered HPA axis are associated with increased systemic inflammation, oxidative stress, and neuroinflammation, impeding CSVD pathophysiology.

### 6.2. Sleep and Gut–Brain Homeostasis: Implications for CSVD and Glymphatic Function

The role of sleep in maintaining gut–brain homeostasis is closely tied to the bidirectional communication within the GBA. Healthy sleep patterns promote the growth of beneficial gut bacteria, which support gut integrity, nutrient absorption, and immune responses. Conversely, poor sleep quality disrupts CNS and gastrointestinal health regulation, i.e., dysbiosis, characterized by the overgrowth of pro-inflammatory bacteria and loss of protective species like *Lactobacillus* and *Bifidobacterium* [73,78]. This imbalance affects gut–brain communication, potentially increasing neuroinflammation and oxidative stress [82].

The glymphatic system, responsible for removing neurotoxic waste products from the brain, such as Aβ, becomes most active during deep sleep; this increases the interstitial space in the brain, thus enabling more efficient removal of these harmful substances from the brain. Poor sleep interferes with this and concurrently decreases the regulation of gut microbiota, which can further enhance neuroinflammatory processes.

Moreover, during sleep, vagal activity (parasympathetic tone) increases, promoting gut motility and the regulation of inflammation. Meanwhile, poor sleep or sleep deprivation dampens vagal tone, impairing gut–brain communication and systemic inflammation, which could accelerate cerebrovascular changes [83]. These results align with the fact that sleep deprivation may weaken the BBB and impair endothelial function, which is important in the pathogenesis of CSVD [73]. Apart from that, poor sleep has also been separately associated with a reduction in the production of SCFAs, further compromising the integrity of the cerebrovasculature and enhancing neuroinflammation [80,81]. The effects of sleep disturbance on the integrity of the cerebrovasculature and neuroinflammation might worsen the outcomes of CSVD by increasing stroke risk, white matter damage, and cognitive impairment. This interrelationship may be viewed as a synergistic relationship emphasising sleep disturbance treatment as integral to preventing CSVD in at-risk populations.

### 6.3. Circadian Regulation by Gut Microbiota and Its Impact on Glymphatic Clearance in CSVD

The emerging evidence outlines that gut microbiota may modulate circadian rhythms, which in turn influence the efficiency of glymphatic clearance, especially during sleep. Gut dysbiosis-coupled circadian rhythm disturbances impede the functions of the glymphatic system, hence leading to an increased risk for CSVD due to the build-up of neurotoxic wastes and increased neuroinflammation. Moreover, the gut microbiota communicate with the host circadian system through microbial metabolites, such as SCFAs and polyamines, whose concentrations oscillate over the course of a day. This communication, in turn, modifies the expression of host circadian genes that regulate sleep–wake cycles, neuroinflammation, and metabolism [84]. For instance, it has been demonstrated that butyrate is an SCFA produced by intestinal microbiota which enhances circadian clock gene expression, such as *Per2*, *Bmal1* and *Clock*, both in the gut and brain, promoting physiological sleep patterns that allow for normal glymphatic activity [85]. Additionally, animal studies also indicate that two *Clostridium sporogenes*-derived metabolites such as 3-(4-hydroxyphenyl)propionic acid (4-OH-PPA) and 3-phenyl propionic acid (PPA) are involved in the regulation of circadian oscillation of *Per2* and *Bmal1* clock genes in the host’s peripheral and central clock machinery, whereby treatment with 4-OH-PPA increased the amplitude and lengthened the period of PER2 oscillation in the suprachiasmatic nucleus and other tissues [86].

Moreover, the glymphatic system, responsible for removing waste from brain metabolic processes, including Aβ, is also optimally active during slow-wave sleep. Microbial dysbiosis contributes to the disrupted production of SCFAs and impairs host circadian entrainment, further contributing to sleep perturbations and reductions in glymphatic clearance. In one recent study, gut dysbiosis resulted in a 25% reduction in SWS, concomitant with a 15% reduction in glymphatic efficiency [87]. This decrease weakens the elimination of neurotoxic waste, creating an environment that enhances neuroinflammation. Enhanced neuroinflammation has been recorded to be a contributor to CSVD pathology. A few microbial metabolites, such as precursors of serotonin and melatonin, have been shown to influence sleep and glymphatic flow. Gut-derived serotonin also contributes to regulating sleep architecture, which in turn indirectly affects the glymphatic process. It has been hypothesised that microbial modulation of serotonin may be responsible for improving sleep quality, enhancing functions of the glial lymphatic system and thus potentially reducing CSVD risk [17,88].

Therefore, the modulation of gut microbiota for restoring circadian rhythm and improving glymphatic function opens new perspectives in preventing CSVD. In this regard, probiotic interventions promoting SCFA production or serotonin pathways may improve sleep quality, reduce neuroinflammation, and enhance glymphatic clearance. However, specific strains and dosages are still under investigation as to how they may restore circadian alignment in patients with dysbiosis. In short, gut microbiota indirectly yet significantly contribute to maintaining glymphatic health through circadian regulation, sleep modulation, and the production of metabolites. Disruption to the system by dysbiosis may disturb glymphatic clearance and increase the risk of CSVD, thus justifying gut-targeted approaches in managing neurovascular health.

### 6.4. Diet’s Influence on Microbiota Composition and Brain Health

Diet, on the other hand, is one of the most important modulators of gut microbiota composition. Food, from fibre and polyphenols to fats and other nutrients, reaches the colon and directly influences microbial diversity, the production of bioactive metabolites, and the overall inflammatory status of the host. For example, high-fibre diets feed bacteria capable of producing SCFAs, including *Bifidobacteria* and *Faecalibacterium prausnitzii*, leading to an anti-inflammatory response and thus promoting vascular and brain health [89]. For example, SCFAs, including butyrate, have been shown to prevent endothelial dysfunction, lower neuroinflammation, and enhance the integrity of the BBB, thus exerting protective effects against CSVD [90].

By contrast, diets high in saturated fat and added sugars are typified by a pro-inflammatory signature of the gut microbiome: reduced gut microbial diversity and a greater abundance of pathogenic bacteria capable of producing pro-inflammatory metabolites, including TMAO. Because TMAO has been strongly associated with atherosclerosis development, vascular dysfunction, and cognitive decline, it is an excellent biomarker of disease risk [62]. Poor dietary habits could promote cerebrovascular damage through inflammation and oxidative stress, thus exacerbating the course of CSVD and impairing the efficiency of brain waste clearance via the glymphatic system, further linking diet to cognitive decline and neurodegeneration [19,91].

Besides macronutrient intake, polyphenols are plant-derived compounds found in fruits, vegetables, and tea that have been recognized to exert beneficial effects on microbiotic composition. A diet rich in polyphenols increases microbial diversity and the abundance of beneficial bacteria, including *Lactobacillus* and *Bifidobacterium*. This boost in the variety and quantity of valuable bacteria helps improve metabolic health, anti-inflammatory effects, and enhanced cognitive function [92]. Interestingly, dietary polyphenols may act as a potential therapeutic candidate to enhance glymphatic function and reduce neuroinflammation, a potential dietary strategy to mitigate the features of CSVD.

### 6.5. The Interplay Between Diet, Sleep, and Gut Microbiota in CSVD

Sleep and diet are two parameters that interact in maintaining gut microbiota and, thus, would affect the risk of CSVD. Poor sleeping habits in combination with a diet characterized by a high intake of processed foods and low fibre intake acts synergistically to promote gut dysbiosis, systemic inflammation, and vascular health impairment [93]. Conversely, the interventions to improve sleep quality and dietary changes by adding fibre and anti-inflammatory nutrients restored microbial balance, reduced systemic inflammation, and promoted good brain health.

Therefore, improving sleep and diet is one of the most promising treatment approaches to prevent and manage CSVD. Indeed, dietary intervention, especially with high fibre and polyphenol intake, has been suggested to positively affect gut microbiota modulation and enhanced glymphatic function to slow down CSVD progression and cognitive decline in support of such intervention [76,80,89]. Additionally, therapeutic interventions that promote sleep, including cognitive behavioural therapy for insomnia (CBT-I) and regulation of the circadian rhythm, will further support gut–brain homeostasis and improve cerebrovascular health [94]. Table 1 summarises the interplay between sleep and diet and their impact on gut microbiota and cerebrovascular health.

The intricate connections between sleep, diet, and gut microbiota underline the importance of integrated therapeutic strategies for managing CSVD. Targeting these modifiable lifestyle factors may offer new avenues for preventing and slowing the progression of cerebrovascular disease. Moreover, given the critical role of inflammation in both CSVD and gut microbiota dysbiosis, it is crucial to explore the role of circulating cell-derived microparticles (MPs) in mediating inflammation and their interactions with the microbiota, as these extracellular vesicles are emerging as key players in the regulation of immune responses and vascular health.

## 7. Role of Microparticles in Inflammation and Microbiota Interaction

### 7.1. Microparticles: Definition and Significance in Cerebrovascular Health

Circulating cell-derived microparticles (MPs) are small membrane-bound vesicles (100–1000 nm in diameter) released by various cell types, including endothelial cells, platelets, and immune cells, in response to cellular activation or apoptosis. MPs play a pivotal role in intercellular communication by transferring bioactive molecules such as proteins, lipids, mRNA, and microRNA between cells and tissues. Their involvement in systemic and cerebral inflammation has garnered significant attention, especially in the context of cerebrovascular diseases like CSVD [95,96]. Elevated levels of MPs have been found in various inflammatory and vascular conditions, making them key contributors to disease progression by promoting oxidative stress, endothelial dysfunction, and thrombogenesis [96].

In CSVD, MPs can directly contribute to cerebrovascular injury by inducing endothelial cell dysfunction, exacerbating BBB disruption, and promoting neuroinflammation. MPs derived from damaged endothelial cells are particularly relevant in CSVD, as they carry pro-inflammatory and pro-coagulant signals that further propagate vascular damage [96]. The clinical relevance of MPs in CSVD is underscored by their presence in higher concentrations in patients with cerebrovascular pathology compared to healthy individuals, making them potential biomarkers for early detection of vascular damage and disease progression [97].

The impact of MPs on cerebrovascular health is multifaceted, particularly when considering their role in inflammation, coagulation, and endothelial function. Recent findings suggest that MPs derived from both endothelial cells and circulating immune cells play a pivotal role in the development and progression of CSVD. For instance, elevated levels of MPs, specifically platelet-derived MP (CD62P), have been detected in individuals with increased risk factors for CSVD, including hypertension, diabetes, and chronic inflammation [97]. MPs contribute to vascular dysfunction by promoting oxidative stress and vascular stiffening, which are hallmark features of CSVD [96]. Additionally, MPs carrying pro-coagulant signals can exacerbate microthrombi formation in small cerebral vessels, further contributing to ischemic damage and white matter lesions [97,98].

Furthermore, despite limited evidence of the role of MPs in glymphatic system function, recent findings on the glymphatic system, particularly in regulating the clearance of neurotoxic waste products such as Aβ, are promising. It is suggested that MPs may influence the activity of astrocytes and the permeability of the BBB, both of which are crucial for glymphatic clearance [13]. Correspondingly, a study by Ruhela et al. reported that astrocyte-derived MPs expressing thrombospondin-1 establish a feed-forward neuroinflammatory cycle involving endothelial CD36-to-astrocyte nuclear factor-kappa beta (NF-κB) crosstalk [99]. This study supports the notion that the dysregulation of MP production or function in the setting of systemic inflammation or sleep disruption may impair glymphatic clearance, contributing to neurodegeneration and cognitive decline, both of which are closely linked to CSVD.

Interestingly, the gut microbiota appear to be a significant modulator of MP-mediated inflammation. In conditions of gut dysbiosis, gut-microbial-derived MPs can exacerbate systemic inflammation, promoting a pro-inflammatory milieu conducive to vascular damage [100,101,102]. Experimental studies have demonstrated that restoring gut microbiota balance through prebiotic or probiotic interventions can reduce the production of pro-inflammatory MPs and improve vascular health [103]. For example, MPs produced by the probiotic *Propionibacterium freudenreichii* CIRM-BIA 129 have been reported to reduce inflammation by modulating the NF-κB pathway [104]. These findings highlight the intricate interplay between gut health, MPs, and cerebrovascular integrity.

### 7.2. Putative Roles of MPs as Mediators of Gut–Brain Communication

Recent research has highlighted the emerging role of MPs in mediating communication between the gut microbiota and the vascular endothelium, impacting both systemic and neuroinflammation. Gut-microbial-derived MPs, particularly those released in response to microbial dysbiosis, can influence vascular health and inflammation by transporting microbial components like LPS and bacterial metabolites into the systemic circulation [105]. Thus, gut-microbial-derived MPs may promote a pro-inflammatory cascade that can exacerbate vascular damage and contribute to the pathophysiology of CSVD.

Gut-microbial-derived MPs have been shown to increase the permeability of the intestinal barrier, facilitating the translocation of LPS into the bloodstream, a process termed metabolic endotoxemia [106]. Elevated levels of circulating LPS and associated MPs trigger systemic inflammatory responses characterized by the release of cytokines like IL-6 and TNF-α, which can also affect cerebral vasculature by enhancing endothelial activation, oxidative stress, and BBB disruption [107]. These interactions are especially relevant to the GBA, where gut-microbial-derived MPs can serve as intermediaries linking gut microbiota dysbiosis to neuroinflammatory processes and cerebrovascular dysfunction.

MPs are also key players in vascular endothelial homeostasis, a crucial factor in maintaining the integrity of cerebral microcirculation. In a dysbiotic state, MPs carrying inflammatory signals may contribute to endothelial dysfunction and impaired glymphatic clearance, further exacerbating CSVD [19]. Dysregulation in the production or clearance of MPs, particularly in inflammatory conditions, has been implicated in the development of neurovascular pathologies, highlighting their potential as both biomarkers and therapeutic targets in diseases like CSVD [108].

MPs are key intermediaries between gut-microbial-derived inflammation and neurovascular health, mainly by amplifying immune responses and promoting endothelial dysfunction. In CSVD, where vascular integrity is already compromised, MPs may serve as both markers of disease progression and potential therapeutic targets. Studies have shown that interventions to reduce MP levels through anti-inflammatory therapies or microbiota-targeted treatments can improve vascular health and reduce the risk of neuroinflammation and cerebrovascular damage [103,104,109]. Table 2 summarizes the multifaceted role of MPs in cerebrovascular disease, linking gut microbiota dysbiosis, systemic inflammation, and CSVD progression.

**Figure 3:**
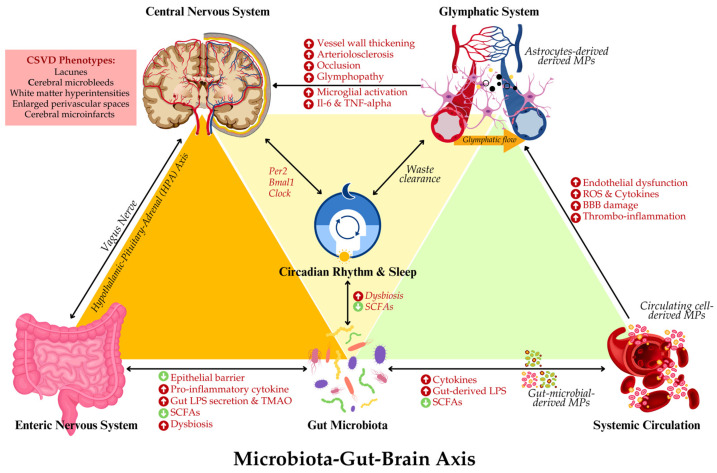
Schematic illustration of the complex interplay between the microbiota–gut–brain axis, cerebral small vessel disease (CSVD), sleep, and the glymphatic system. Dysbiosis in gut microbiota triggers inflammatory responses, producing cytokines, lipopolysaccharides (LPS) and reducing short-chain fatty acids (SCFAs) that affect the central nervous system (CNS). Disruptions in circadian rhythm impair glymphatic waste clearance, impacting glymphatic flow and microglial activity. Gut-microbial-, circulating cell- and astrocyte-derived microparticles (MPs) and inflammatory mediators contribute to blood–brain barrier (BBB) damage and thrombo-inflammation. These interactions promote CSVD phenotypes, including microbleeds and white matter changes, emphasizing the role of systemic inflammation and neuroimmune dysfunction in CSVD progression.

These findings underscore the significance of MPs as contributors and mediators in the pathogenesis of CSVD. Their role in facilitating gut–brain communication, particularly in the context of inflammation, highlights their potential as therapeutic targets for mitigating the effects of gut dysbiosis and cerebrovascular dysfunction. Therefore, given the significant role of MPs in modulating inflammation and vascular health, it is critical to explore therapeutic strategies that aim to modulate the microbiota to improve neurological health. Figure 3 summarises the putative interrelationship between GBA-microbiota, MPs and glymphopathy. The following section will delve into the potential of microbiota-targeted interventions as a novel therapeutic approach for managing CSVD and neuroinflammation.

## 8. Therapeutic Potential: Modulating Microbiota for Neurological Health

This new link between gut microbiota and cerebrovascular integrity through neuroinflammation opens a window of opportunity for therapeutic intervention. Current strategies include probiotics, prebiotics, dietary manipulations, and FMT as effective ways to modulate gut microbiota to reduce inflammation, enhance vascular health, and improve glymphatic clearance. More recently, attention has been paid to lowering the burden of MPs on systemic and cerebrovascular inflammation.

### 8.1. Probiotics, Prebiotics, and Dietary Interventions

Probiotics are living microorganisms that, when administered in adequate amounts, provide health benefits to the host by enhancing microbial balance. Some probiotics, such as *Lactobacillus rhamnosus*, *Bifidobacterium longum*, and *Akkermansia muciniphila*, show neuroprotective effects via immunomodulatory response, gut barrier integrity, and reduction of systemic inflammation [110,111]. They have been shown to be effective in animal models of CSVD, improving cognitive outcomes and blunting vascular dysfunction by producing SCFAs and anti-inflammatory cytokines [112].

*L. rhamnosus* and *B. longum* are the most well-recognized and highly studied probiotic species with neuroprotective effects. Recent publications indicate that *L. rhamnosus*, by modulating neuroinflammation via the GBA, resulted in a significant reduction in systemic markers of inflammation, represented by IL-6 and TNF-α, in LPS-induced *Escherichia coli*-driven inflammation in C57BL/6 mice [113]. *B. longum,* in turn, is observed to promote endothelial health by increasing nitric oxide bioavailability, enhancing vascular dilation, and thereby decreasing blood pressure, a feature particularly pertinent to the management of CSVD [114]. The translation of such findings into human populations has been variable, as microbial composition and host factors, including age or diet, influence the efficacy of such probiotics. To this date, inconsistent clinical trial results have emerged, with only approximately 60% of patients showing detectable neuroinflammation reductions [115].

Probiotic supplementation has been associated with reduced circulating levels of TNF-α, IL-6, and CRP [116], all of which are increased in CSVD phenotypes. By improving gut barrier integrity and reducing pro-inflammatory cytokine production, probiotics may prevent the translocation of microbial endotoxins, which are linked to neuroinflammation and endothelial dysfunction. Of particular note, *Akkermansia muciniphila* has emerged as a novel probiotic with demonstrated benefits for endothelial function via enhancement of gut barrier integrity and reduction of systemic endotoxemia. Animal studies have postulated that it reduces neuroinflammatory cytokines by more than 20%, reducing vascular inflammation markers such as IL-6 [117]. However, the strain’s clinical efficacy in humans remains limited by its low natural abundance in Western diets, requiring high doses for measurable effects. Other roles of SCFAs relate to the modulation of cerebral blood flow and glymphatic clearance, which is very important as SCFA-producing bacteria such as *Butyrivibrio fibrisolvens* take part in maintaining the integrity of cerebral vasculature [118].

One study investigates the relationship between gut microbiota, sleep quality, and cognitive flexibility in older adults, shedding light on how these factors interact in age-related cognitive resilience [119]. The stool samples of 37 healthy older adults were analysed along with the assessment of sleep quality and cognitive flexibility by the Stroop test. Results indicated that higher sleep quality is related to better Stroop performance and a higher relative abundance of the gut microbial phyla Verrucomicrobia and Lentisphaerae. Interestingly, the relation of cognitive flexibility with Lentisphaerae was partly mediated by sleep quality [119]. This moderative effect may suggest that sleep is also an important factor through which the gut–brain axis modulates cognitive flexibility. The influence of Verrucomicrobia was primarily linked to sleep quality. Consequently, it is still unknown whether Verrucomicrobia directly impacts cognitive measures. These findings underlined the potential of gut microbiota as one such contributor to cognitive flexibility in older adults, influenced largely by sleep quality. Further studies should investigate whether strategies to improve gut microbiome health might protect against sleep-related cognitive decline. The findings open a way for exploring microbiome-modulating treatments; however, experimental approaches are also needed to verify these preliminary results and clinically test potential therapeutic interventions for ageing populations.

Conversely, prebiotics are indigestible food ingredients that stimulate beneficial bacterial growth and play an essential role in modulating the GBA. Prebiotic fibres such as inulin and fructo-oligosaccharides stimulate the proliferation of SCFA-producing bacteria, including *Faecalibacterium prausnitzii*, which play a role in immune modulation and endothelial health and enhance glymphatic clearance and reduce oxidative stress in the brain [120]. Yet, despite these promising effects, prebiotics induce gastrointestinal discomfort—such as bloating or diarrhoea—which certainly limits the compliance and long-term use of these substrates in the clinical setting. Intriguingly, evidence is emerging that prebiotics improve endothelial function and reduce vascular stiffness [121], an important aspect of managing CSVD.

Moreover, diets rich in fibre, polyphenols, and omega-3 fatty acids can be considered as dietary interventions targeting the microbiota. High-fibre diets increase the production of SCFAs, whereas polyphenolic compounds such as flavonoids and resveratrol have been reported to affect gut microbiota composition, lowering oxidative stress and improving vascular health [122]. Besides, omega-3 fatty acids, especially eicosapentaenoic acid (EPA) and docosahexaenoic acid (DHA), may act directly on gut microbiota composition, increasing the abundance of anti-inflammatory bacterial strains while decreasing vascular inflammation [123,124], i.e., the risk for CSVD. Therefore, Table 3 summarises the potential therapeutic approaches to modulating gut microbiota for CSVD and glymphatic function.

Large heterogeneity in gut microbiota among individuals prevents the extrapolation of those findings into the clinical setting due to different individual responses to the administration of probiotics and prebiotics. Further, the absence of standard probiotic strains and dosing makes it even more challenging to reproduce the same effect in different studies. Only minimal data are present on long-term safety, especially for newer probiotics like *A. muciniphila*, and its cautious application needs to be defined in CSVD patients. While some are promising, including certain probiotics and prebiotics for neuroinflammation and vascular health, it is variability from host responses that places limitations on the current clinical translatability due to logistical issues related to dosing and tolerability. Further research into strain-specific effects is needed, with the development of uniform formulations that would contribute to the optimization of such interventions in the management of CSVD.

### 8.2. Faecal Microbiota Transplantation (FMT) and Emerging Therapies

The FMT is one of those rising stars that has gained increasing attention and turned into an intervention for restoring microbial diversity and decreasing inflammation in disrupted gut microbiota. FMT was successfully applied to patients with recurrent *Clostridium difficile* infections [125], while in the case of application for neurological diseases, including CSVD, interest has been growing [126]. Indeed, various pre-clinical and human studies have shown the potential of FMT to dampen neuroinflammation and improve cognitive function, even to the restoration of glymphatic clearance of toxic proteins such as amyloid-β [127,128].

One of the main mechanisms through which FMT would benefit subjects with CSVD would be the restitution of healthy microbiota that may increase the production of SCFAs and decrease systemic inflammation. FMT also directly restored the integrity of the intestinal barrier, preventing translocation into the systemic circulation of proinflammatory bacterial products, including LPS, a process intimately linked to neuroinflammation and endothelial dysfunction [125]. Further, newer microbiota-based therapies such as postbiotics and synbiotics have also been explored for neuroprotective potential [129]. Non-viable bacterial products or metabolic byproducts, such as SCFAs or postbiotics, have effectively lowered systemic inflammation and promoted vascular health [130]. Synbiotics are a combination of probiotics and prebiotics that have been studied in relation to their synergetic effects on GBA functioning, with initial positive results in lessened cognitive decline in animal models with vascular dementia [129].

Given this context, gut microbiota-associated metabolites are emerging as pivotal regulators in disease progression and promising therapeutic agents due to their natural bioavailability, high concentrations, ease of administration, and tissue tolerability [130,131]. Such metabolites, including trimethylamine N-oxide (TMAO), have been implicated in the pathology of cardiovascular disease, although their functional role remains highly debated between detriments and protection [62]. SCFAs, such as butyrate, have anti-inflammatory properties, improving symptoms of brain-associated inflammatory conditions, autoimmune diseases like multiple sclerosis [132], and stress-related mood disorders [133]. Recent studies also highlight the antiviral potential of microbiota-derived metabolites against COVID-19 and the possible benefits of FMT for symptom relief [134]. Besides, there is an advance in the study of metabolomics that proves the involvement of gut microbiota metabolites in colorectal cancer: preclinical and clinical studies with butyrate supplementation prevented tumour development and improved radiotherapy efficiency [135]. These findings underscore the clinical potential of gut microbiota-derived metabolites in treating various diseases.

### 8.3. Strategies to Control the Levels of MPs

Anti-inflammatory interventions directed at the GBA or direct inhibition of the formation of MPs by pharmacological agents, including statins and angiotensin-converting enzyme (ACE) inhibitors, represent potential therapeutic strategies to reduce MPs [96]. Reduction of circulating MPs has indeed been associated with an improvement in vascular function and reduction of ischemic stroke risk [136], in particular in patients affected by CSVD. Furthermore, novel anti-inflammatory therapies target major pathways involved in neurovascular inflammation, such as MPs, especially in CSVD. For example, the use of proinflammatory cytokines (i.e., TNF-α, IL-1β, and IL-6) inhibitors and neutralising antibodies may serve as potential target therapies for reducing MP secretions. This can be achieved by blocking the pro-inflammatory cytokine activity and inhibiting inflammatory mediators, such as cyclooxygenase-2 (COX-2). For example, statins are renowned not only for cholesterol-lowering but also for anti-inflammatory action. They have also been reported to reduce vascular inflammation and oxidative stress [137]. This brings about the controlling effect of MPs on vasculature and, thus, improves endothelial function in CSVD patients.

ACE inhibitors and angiotensin II receptor blockers (ARBs) represent another pharmacological strategy aimed at reducing vascular inflammation by inhibiting the renin–angiotensin system, implicated in both hypertension and vascular dysfunction [138], i.e., risk factors for MPs formation (and vice versa) and common features of CSVD. Specific probiotics have been proven to modulate MPs release by reducing endothelial activation and inflammation, indicating a possible microbiota-targeted strategy to control MP-related vascular damage [103,104,109].

While such microbiota-targeting therapies herald encouraging prospects, challenges and limitations exist in the path towards these therapies. First, the gut microbiota are considerably heterogeneous among individuals, yielding variability in therapeutic responses [139]. Personalized approaches that would consider individual microbiota profiles and genetic backgrounds are likely needed to optimise therapeutic benefits. However, standardization of probiotics, prebiotics, and FMT remains one of the main challenges in their clinical use. The fact that probiotic strains differ from one another, and have various effects on microbiota and the immune response, makes generalization difficult among studies. In the same way, the use of FMT in neurological diseases has not yet been established in terms of long-term safety or efficacy, raising concerns of possible adverse effects and durability of microbiota changes.

Future multi-modal treatments will include combinations of microbiota-targeted therapies with other anti-inflammatory or neuroprotective compounds that could bring synergistic benefits in patients with CSVD. Future improvements in metagenomic analysis and profiling technologies will also allow more precise identification of microbial species and metabolites implicated in the GBA and enable more selective therapeutic strategies [140]. While the knowledge of gut microbiota interaction with neuroinflammation and vascular health becomes more advanced, so do microbiota-based therapies for preventing and treating CSVD and other neurovascular disorders. Consequently, this will lead to a comprehension of how combining microbiotic modulation with newly developed anti-inflammatory strategies will further improve neurovascular health and enhance clinical outcomes in patients with CSVD.

Combining microbiotic modulation with emerging anti-inflammatory strategies might lead to a synergistic approach that will significantly enhance neurovascular health and improve clinical outcomes in CSVD patients. This approach leverages the GBA, where the gut microbiota play a key role in regulating systemic inflammation, including that mediated by MPs, affecting vascular health and neurological function. Clinicians would thus be able to address the multifactorial pathogenesis of CSVD, involving vascular dysfunction, endothelial damage, oxidative stress, and neuroinflammation, by targeting both the microbiota and inflammation.

### 8.4. Synergistic Effects: Microbiotic Modulation Combined with Anti-Inflammatory Therapy

Firstly, SCFA production and immune modulation are improved. Microbiota-targeted interventions, including the use of probiotics and prebiotics, increase SCFA production, which in turn helps reduce systemic inflammation and oxidative stress. SCFAs directly influence the immune system by enhancing anti-inflammatory cytokine production, such as IL-10 and Treg cells that modulate inflammation locally in the gut and systemically [141]. When these approaches are combined with anti-inflammatory strategies, either statins or COX-2 inhibitors, the total inflammatory burden would be profoundly decreased, with actions of both stabilizing endothelial function and reducing the neurovascular damage typical in CSVD.

The second mechanism is through the reduction of circulating MPs. As discussed, CSVD patients have reported increased levels of circulating MPs, released after vascular and endothelial injury, closely associated with increased inflammation and vascular dysfunction. Targeting the microbiota with probiotics and prebiotics reduces MP release by modulating endothelial cell activation. Coupling the latter approach with anti-inflammatory agents such as statins, known to reduce MP levels, may improve therapeutic efficacy by reducing ischemic events and improving vascular health [142].

Next is the integrity of the gut barrier and BBB protection. Gut dysbiosis and the resulting increased intestinal permeability (leaky gut) drive systemic inflammation by allowing bacterial components, such as LPS, to leak into the bloodstream and thus induce neuroinflammation [103]. Pure anti-inflammatory therapies might not suffice to address the origin of the inflammation. However, restoring gut barrier function by microbiotic modulation may prevent the translatability of harmful bacterial products across the barrier and reduce the systemic inflammatory burden. This restoration may exert a dual effect—for instance, the reduction of gut-derived inflammation can directly inhibit the pro-inflammatory pathways in the brain either through drugs such as inhibitors of IL-6 or blockers of TNF-α, and therefore, this combined strategy could enhance the integrity of the BBB and protect against further damage to the brain.

Modulation of oxidative stress and function of the glymphatic system have also been referred to as synergistic effects. Oxidative stress acts as a key player in endothelial dysfunction and cerebral microvascular damage due to CSVD. Probiotics reduce markers of oxidative stress, strains of *Lactobacillus* and *Bifidobacterium* [143], and anti-inflammatory drugs, such as statins, exhibit antioxidant actions through their effects on nuclear factor erythroid 2-related factor 2 (Nrf2) [144]. Such a double-edged strategy may improve vascular function and glymphatic elimination of noxious waste products, including Aβ accumulating in the neurodegenerative disorders associated with CSVD.

Finally, there is the circadian rhythm and glymphatic regulation. Gut microbiota are critical in regulating the circadian rhythm and vital for glymphatic clearance, especially during sleep. A disruption in this circadian rhythm is associated with glymphopathy and increased neurovascular damage. Anti-inflammatory therapies that reduce sleep disturbances and enhance vascular health may act synergistically with microbiota-targeted interventions that improve circadian regulation and glymphatic function. Such may be the case, for example, with probiotics targeting melatonin production or sleep quality acting in concert with anti-inflammatory drugs that target neurovascular inflammation [145,146].

## 9. Clinical Implications and Future Directions

A combined approach of microbiotic modulation with anti-inflammatory therapies may significantly improve the clinical outcome in CSVD patients. Although favourable results have emerged from preclinical studies and early clinical trials, more extensive and well-designed randomised control trials (RCTs) must be conducted to confirm such findings. One of the critical challenges in that respect concerns the heterogeneity within the gut microbiota across individuals. Thus, future therapies may have to be personalized depending on the individual’s microbiome profile, genetic makeup, and disease status. Long-term safety and efficacy need to be established for microbiota-targeted therapies, especially for FMT and postbiotics, before wide clinical acceptance of these approaches can be achieved.

Moreover, establishing biomarkers that monitor the effect of both microbiota and anti-inflammatory treatments in real time will improve the precision of treatment. Such biomarkers might include microbial metabolites like SCFAs, inflammatory cytokines, and circulating MPs that give insight into the efficacy of applied treatments and enable early intervention in CSVD patients.

In brief, the integration of microbiotic modulation with new anti-inflammatory strategies thus provides a more holistic approach to the management of CSVD. Such an integrated strategy may better address inflammation and microbial contributors to neurovascular dysfunction and, as such, may potentially reduce disease progression and improve glymphatic clearance in patients with CSVD.

## 10. Conclusions

The complex interaction between gut microbiota, cerebrovascular health, and neurological function makes microbiota important modulators of CSVD. Dysbiosis promotes systemic inflammation, neuroinflammation, and vascular dysfunction which accelerate CSVD. Emerging evidence underlines the role of the GBA in modulating neurovascular integrity, particularly through the interactions with the glymphatic system and circulating MPs. Target modulation by probiotics, prebiotics, and dietary interventions presents a promising therapeutic strategy for restoring gut–brain homeostasis, enhancing waste clearance, and reducing neurovascular damage. This strategy might be usefully combined into anti-inflammatory treatments, opening a new avenue for mitigating CSVD and improving cognitive outcomes. As research continues, personalized microbiome-based interventions may offer novel strategies for preventing and treating neurovascular disorders, potentially leading to better long-term brain health. Future studies should be directed at validating such therapeutic approaches and exploring their clinical benefit in managing CSVD.

## Figures and Tables

**Table 1:** Impact of sleep and diet on gut microbiota and cerebrovascular health.

Factors	Effects on Gut Microbiota	Impact on Cerebrovascular Health
Sleep deprivation	Reduced microbial diversity Increased pro-inflammatory bacteria	Impaired glymphatic clearance Increased neuroinflammation Weakened blood–brain barrier
High-fibre diet	Increased short-chain fatty acid (SCFA)-producing bacteria (e.g., *Bifidobacteria* sp.)	Reduced vascular inflammation Improved endothelial function
High-fat diet	Increased trimethylamine N-oxide (TMAO)-producing bacteria	Elevated TMAO levels Increased risk of atherosclerosis and cognitive decline
Poor sleep and diet	Synergistic effect on dysbiosis and inflammation	Exacerbates cerebral small vessel disease (CSVD) Cognitive impairment Vascular dysfunction
Polyphenol-rich diet	Enhanced microbial diversity Increased *Lactobacillus* sp.	Reduced neuroinflammation Enhanced glymphatic function

**Table 2:** Microparticles (MPs) in gut–brain interaction and cerebrovascular health.

MPs Source	Mechanism of Action	Impact on CSVD
Circulating cell-derived MPs (e.g., platelets and endothelial cells-derived)	Promote oxidative stress Increase coagulation	Endothelial dysfunction Impaired blood–brain barrier (BBB) Increased microthrombi formation and accumulation
Gut-microbial-derived MPs	Carry lipopolysaccharides (LPS) Carry microbial metabolites	Systemic inflammation Vascular endothelial dysfunction
Immune cell-derived MPs	Trigger pro-inflammatory cytokines release	Neuroinflammation BBB disruption
Probiotic-regulated MPs	Restore gut barrier integrity	Reduced inflammation Improved vascular health

**Table 3:** Therapeutic approaches to modulating gut microbiota for CSVD and glymphatic function.

Therapy	Mechanism	Potential Impact on Glymphatic System & CSVD
Probiotics	Modulate immune response Enhance short-chain fatty acids (SCFAs) production	Reduce neuroinflammation Improve endothelial function
Prebiotics	Stimulate SCFA-producing bacteria	Reduce systemic inflammation Enhance glymphatic clearance
Omega-3 Fatty Acids	Modulate gut microbiota Reduce oxidative stress	Improve vascular health Reduce cerebral microvascular damage
Polyphenol-Rich Diet	Anti-oxidative and anti-inflammatory effects on gut bacteria	Protect against vascular dysfunction Reduce oxidative stress

## Data Availability

Not applicable.
